# Broadening understanding of accountability ecosystems in sexual and reproductive health and rights: A systematic review

**DOI:** 10.1371/journal.pone.0196788

**Published:** 2018-05-31

**Authors:** Sara Van Belle, Vicky Boydell, Asha S. George, Derrick W. Brinkerhof, Rajat Khosla

**Affiliations:** 1 Institute of Tropical Medicine, Antwerp, Belgium; 2 The Evidence Project, International Planned Parenthood Federation, London, United Kingdom; 3 School of Public Health, University of the Western Cape, Cape Town, South Africa; 4 RTI International, Research Triangle Park, North Carolina, United States of America; 5 World Health Organization, Geneva, Switzerland; TNO, NETHERLANDS

## Abstract

**Background:**

Accountability for ensuring sexual and reproductive health and rights is increasingly receiving global attention. Less attention has been paid to accountability mechanisms for sexual and reproductive health and rights at national and sub-national level, the focus of this systematic review.

**Methods:**

We searched for peer-reviewed literature using accountability, sexual and reproductive health, human rights and accountability instrument search terms across three electronic databases, covering public health, social sciences and legal studies. The search yielded 1906 articles, 40 of which met the inclusion and exclusion criteria (articles on low and middle-income countries in English, Spanish, French and Portuguese published from 1994 and October 2016) defined by a peer reviewed protocol.

**Results:**

Studies were analyzed thematically and through frequencies where appropriate. They were drawn from 41 low- and middle-income countries, with just over half of the publications from the public health literature, 13 from legal studies and the remaining six from social science literature. Accountability was discussed in five health areas: maternal, neonatal and child health services, HIV services, gender-based violence, lesbian/gay/bisexual/transgender access and access to reproductive health care in general. We identified three main groupings of accountability strategies: performance, social and legal accountability.

**Conclusion:**

The review identified an increasing trend in the publication of accountability initiatives in Sexual and Reproductive Health and Rights (SRHR). The review points towards a complex ‘accountability ecosystem’ with multiple actors with a range of roles, responsibilities and interactions across levels from the transnational to the local. These accountability strategies are not mutually exclusive, but they do change the terms of engagement between the actors involved. The publications provide little insight on the connections between these accountability strategies and on the contextual conditions for the successful implementation of the accountability interventions. Obtaining a more nuanced understanding of various underpinnings of a successful approach to accountability at national and sub national levels is essential.

## Introduction

Accountability has long been a key theme in international development and its related disciplines [1–2]. For health systems specifically, accountability lies at the heart of how power relations in service delivery are negotiated and implemented, whether framed by those in the women’s health movement [3] or by those from multilateral lending organisations [4]. It is also at the core of applying human rights to development and health, whether through their incorporation in economic development [5]; or in preventing and redressing human rights violations [6], or in the monitoring of human rights treaties applied to health [7–8].

Recently, accountability in health has become a key priority at the highest levels of the United Nations system through its engagement with national governments. The Commission on Information and Accountability for Women’s and Children’s Health (CoIA), founded in 2010 as a follow-up to the UN Secretary General’s initiative “Every Woman, Every Child”, recommended that all countries establish and strengthen accountability mechanisms that are transparent and inclusive of all stakeholders [9]. This was reiterated by the Independent Expert Review Group (iERG), which called for strengthening of human rights instruments to improve accountability for women’s and children’s health. The iERG recommended that health ministries prioritise national oversight mechanisms to advance women’s and children’s health with non-state partners at country level [10]. This is echoed in the new Global Strategy on Women’s, Children’s and Adolescents’ Health (2016–2030), whereby accountability is recognised as a key action area that harmonizes monitoring and reporting; improves civil registration and vital statistics; and promotes independent review and multi-stakeholder engagement [11]. In 2016, as part of a unified accountability framework, the first report of the Independent Accountability Panel (IAP) further highlighted the need to strengthen rights-based accountability at the national level [12].

Despite increased attention to and demand for accountability in health from multiple and varied global stakeholders, understanding of accountability initiatives for sexual and reproductive health at national and sub-national levels remains limited. Given the multi-disciplinary contributions to understanding accountability, we undertook a systematic review of peer-reviewed literature across disciplinary boundaries. Considering this complexity, at an initial stage in our systematic review, we sought to map the range of accountability strategies and instruments used to address sexual and reproductive health and rights, the low and middle-income contexts in which they were implemented and the resulting documented outcomes.

In the paper, we use the terms “accountability strategy”, “accountability intervention”, “accountability instrument” and “accountability mechanisms”.

- An “accountability strategy” is any overarching set of programmes and activities, conducted by governments, non-governmental organizations (NGOs), grassroots organizations, activist lawyers as well as communities with the intention to enforce or support accountability.
- The term “accountability intervention” refers more narrowly to the operational level. Examples include setting up a village health committee, bringing a court case or carrying out a drama workshop to educate villagers on sexual and reproductive health rights (SRHR). Interventions are usually delivered within projects or programmes with the objective of supporting accountability.
- An “accountability instrument” is the use of particular implementation tools within the context of a given intervention. Examples include patient charter rights or digital health feedback applications.
- An “accountability mechanism” is a theoretical explanation of why a strategy or intervention works. Explanatory theoretical mechanisms include collective action, community empowerment, transparency, and enforcement.

## Methods

The review methodology was initially structured with a realist and multi-disciplinary intent to ask “what works in terms of accountability mechanisms in the field of sexual and reproductive health rights (SRHR) at sub-national and national levels, how, why and in which context?”. The review is based on a protocol that was reviewed by an international expert technical committee. We were guided by a meta-interpretation approach [13], which maintains an interpretive epistemology in its analysis, congruent with primary qualitative research. The guiding principles of meta-interpretation are (1) avoiding predetermined exclusion criteria; (2) a focus on meaning in context; (3) using interpretation as unit for synthesis; (4) an iterative approach to theoretical sampling of the studies, and (5) a transparent audit trail to ensure the integrity of the synthesis. It is suitable for this review because it allows capturing the different dimensions relevant to accountability strategies at national and sub-national levels relevant to SRHR accountability.

### Search strategy

To capture the accountability strategies across multiple disciplines, we used three search engines: PubMed (health literature), Web of Knowledge (social sciences) and LexisNexis Academic (law). The search terms included combinations of free-text words in TI and /or all fields, depending on the search strategies allowed by the database in question (Table 1 and S1 Table). We refer to the latter for the Boolean operators used for each database search strategy.

**Table 1:** Search terms.

Accountability terms	Accountability / accountable (noun/adjective), (public) accountability, (community) accountability, (social) accountability, answerability, enforcement
Sexual and Reproductive Health Terms	(Gender-based, sexual, domestic) violence, maternal mortality, maternal morbidity, sexually transmitted infection (STI), HIV, (unintended, unwanted, teenage) pregnancies, (unsafe) abortion, adolescent sexual and reproductive health, adolescent sexual and reproductive rights, obstetric care, respectful childbirth, referral, antenatal care, contraception, family planning, infertility, prevention of mother to child transmission of HIV (PMTCT), perinatal mortality, perinatal morbidity, fistula, abuse, female genital mutilation (FGM), child marriage
Human rights-sexual and reproductive rights terms	Equality, equity, stigma, non-discrimination, accountability, privacy and confidentiality, informed decision-making, participation, availability, accessibility, acceptability, quality of care, sexual rights, reproductive rights, sexual and reproductive rights, sexual and reproductive health and rights, right to health, women's rights, lesbian-gay-bisexual-transgender (LGBT) rights, intersex rights, respect, disrespect
Accountability Instruments- Terms	Parliamentary commissions, civil service ombudsman, professional associations, commission on administrative justice, right to information act, consumer forums, health committees, ombudsman services, health commissioners, citizen score cards, right to information, Constitution, annual health summit; public investigators; health sector review; health councils/hospital boards; professional associations (accreditation); health committees; patient/user groups; patients charter; audit bodies; budget committees; ombudsman

Options to select languages other than English were limited in the three databases. In LexisNexis Academic, two categories of law reviews were available to cover different languages: (1) UK and European journals and (2) Brazilian, Asian law and French language journals and reviews. The UK/European law journals also include journals on legal traditions from LMIC, e.g. Journal of African Law and the Journal of Asian Law. No specific language or country selection options could be made in PubMed and Web of Science.

### Study selection

Each abstract was screened using the inclusion and exclusion criteria presented in Table 2, covering time period, geographic range, language and publication type.

**Table 2:** Inclusion and exclusion criteria.

Criteria	Included	Excluded
**Timeline**	1994—October 30, 2016	Before 1994
**Countries**	Low-and Middle-Income Countries as per Organisation for Economic Cooperation and development (OECD) Development Assistance Committee (DAC) List of Overseas Development Assistance (ODA) Recipients	All other countries
**Languages**	English, Spanish, French, Portuguese	All other languages
**Publication type**	Empirical studies / primary data analysis: randomized control trials; quasi-experimental studies, before/after, longitudinal and qualitative studies (e.g. case studies, action research, grounded theory, ethnography) Articles in academic law journals, academic law reviews Systematic reviews (all types) Comments, critical reflections presenting empirical case-studies to illustrate	Non-peer reviewed empirical studies NGO Meeting reports NGO programme reports NGO advocacy publications Conference proceedings Dissertations On-going research Protocols (NGO and other) Programme evaluations, or programme reports with an evaluative component Comments, expert opinion or reflections with an evaluative component Book reviews

The abstracts that met the inclusion criteria (articles on low and middle income countries in English, Spanish, French and Portuguese published from 1994 onwards—the year of the first International Conference on Population and Development was organised publications on low and middle income countries) were then reviewed to assess if they (1) relate to any accountability strategy or mechanism, (2) relate to a SRHR area or (3) a national level judicial or reconciliation mechanism (such as court proceedings of international war tribunals). The latter included studies reviewing jurisprudence from supreme, constitutional or other national and provincial level courts. To verify fidelity to the inclusion criteria, a sample of 20 abstracts per database were checked for inclusion/exclusion by a second senior researcher. Two researchers discussed the papers for which they had a different opinion until a consensus was reached.

After the full text review, articles were further excluded based on the exclusion criteria (e.g. articles related to global and regional accountability mechanisms). We present the papers that were included in S2 Table.

### Data extraction

The review question guided the data extraction. Categories included in the data extraction include: (1) author; (2) SRHR issue; (3) year of publication; (4) number of citations (Google Scholar); (5) year of intervention; (6) original language; (7) funding source; (8) study setting; (9) type of study; (10) accountability type according to the article or as deduced by the researcher; (11) accountability relationship (from whom to whom); (12) accountability strategy and implementation instrument; (13) level at which strategy is supposed to work; (14) purpose (why?); (15) lessons learned; (16) reported outcomes; (17) mechanisms; (18) equity effects; (19) description of the intervention or action; (20) scale of the intervention or action; (21) target population and finally (22) the actors involved in the accountability strategy.

### Data analysis

Since this review covers several disciplines (public health, social sciences, legal studies) with different disciplinary standards for writing and quality appraisal, it is difficult to apply a single framework to assess quality across the cases. Legal reviews, for instance, apply a critical (post-positivist) paradigm and typically do not provide a methodology section. Other studies included do not neatly distinguish between reporting and interpreting results. To gauge quality across the papers, we applied the principles of data quality appraisal for qualitative research [14] and used “Not applicable (NA)” when criteria were not applicable (see S3 Table).

Narrative synthesis [15] was used to summarize results and numerical frequencies per category were calculated, whenever this was applicable. Thematic analysis was used to examine the different categories of accountability strategies that emerged.

## Results

### Study selection

**Fig 1:**
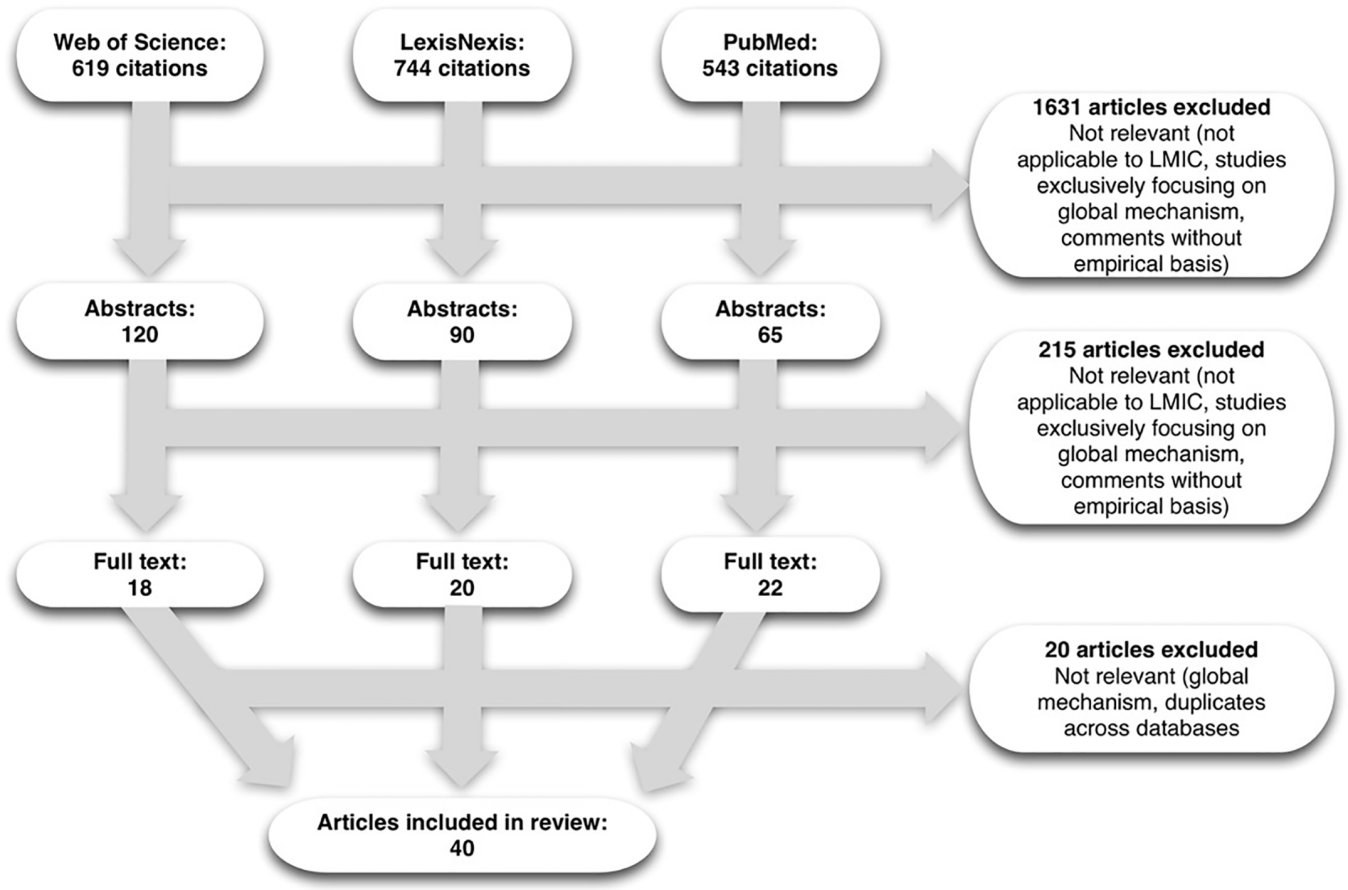
The PRISMA flowchart.

A total of 1,906 articles were found when the search terms were applied to the three databases. On application of the inclusion and exclusion criteria, 1,631 abstracts were excluded. Further review of the 275 included abstracts led to sixty articles downloaded for a full-text review: 18 articles were retained from Web of Science, 20 articles from LexisNexis Academic, and 22 from PubMed. The articles came from public health, legal studies, political science, history, social psychology, anthropology, critical theory, ethics, health services management, clinical sciences, public administration, conflict studies, transitional and restorative justice studies, development and humanitarian studies. This underscores the need for an interdisciplinary approach to understand and examine the different aspects related to accountability in health. After the full text review, twenty out of sixty articles were further excluded resulting in the final selection of 40 articles documenting experiences related to accountability for SRHR at national and subnational level in low and middle-income countries between 1994–2016 (Fig 1 The PRISMA flowchart, S4 Table and S1 File).

### Study characteristics

Of the 41 low- and middle-income countries featured, eighteen articles reported on cases in sub-Saharan Africa, 10 in Latin America, nine in Asia, three in the Middle East/Maghreb and one in Europe. Several countries were represented in multiple articles: India (6 studies), South Africa (5 studies), Nigeria (3 articles) and Guatemala (2 studies). Seven studies were in humanitarian or post-conflict settings (Somaliland, Afghanistan, Haiti, Sierra Leone, Liberia, Guatemala and Peru). Nine articles reported on multi-country interventions or used examples from more than one country.

While the search period ran between 1994 and October 2016, the majority of articles were published between 2014 and 2015. A range of disciplines and study designs are included (Table 3). Just over half (21 of the 40) of the publications were found among the literature on public health, and a significant number (13) were found from legal studies. Six were drawn from the social sciences (anthropology, political science, development studies and sociology). While over half were qualitative case studies, only two ethnographies, one action research and two critical study articles were found.

**Table 3:** Research design by SRHR area.

Research design	Maternal, Neonatal and Child Health (MNCH)	HIV	Gender-based violence	LGBT access	Reproductive health care in general	Row Total
**Systematic review**	Pattinson et al., 2009 [16]	0	0	0	0	1
**Cross-sectional**	Asefa & Bekele, 2015 [17], Rosen et al., 2015 [18]	0	0	0	0	2
**Case studies, qualitative**	Papp et al. 2013 [19], Ding 2015 [20], Hulton et al. 2014 [21], Mafuta et al. 2015 [22], Shayo et al. 2013 [23]	McPherson et al. 2013 [24], Topp et al. 2015 [25] Tromp et al 2015 [26]	Bendana & Chopra 2013 [27]	0	0	9
**Descriptive studies**	Freedman 2003 [28], Garba & Bandali 2014 [29], Mathai et al. 2015 [30], Hussein & Okonofua 2012 [31], Scott & Danel 2016 [32], Ouédraogo et al. 2014 [33], Labrique et al. 2012 [34], Ghosh 2011 [35]	0	Seelinger 2014 [36], Barrow 2009 [37]	0	0	10
**Policy analysis**	Blake et al. 2016 [38], George 2003 [39]	0	0	Penas Defago & Moran Faundes 2014 [40]	0	3
**Ethnography**	Behague et al., 2008 [41]	0	0	McCrudden 2015 [42]	0	2
**Legal reviews**	Kaur 2012 [43]	Durojaye & Balogun, 2010 [44]	0	Khaitan 2015 [45], Miles 2015 [46]	Chirwa 2005 [47], Davis 2008 [48], Nolan 2014 [49], Orago 2015 [50]	8
**Action research**	0	0	Crosby & Lykesy, 2011 [51]	0	0	1
**Critical studies**	0	0	0	Lind & Keating 2015 [52]	Rinker, 2015 [53]	2
**Undefined**	0	0	Duggan et al. 2008 [54], Du Toit 2016 [55]	0	0	2
**Column total**	20	4	6	5	5	40

In terms of study quality, we found that eighteen papers presented an audit trail, 15 had a sampling process described, and in 15 papers, triangulation, member checking or deviant case analysis was used to ascertain validity. Fourteen out of 40 studies (35%) obtained the highest score for explanatory power, only 6 (15%) obtained the highest score for insider comprehensiveness, 13 (32,5%) did so for the advancement of knowledge and 5 out of 40 studies (12.5%) for detail (i.e. making the study clear for outsiders) (see S5 Table). Eighteen out of 40 studies (45%) displayed some proof of long-term field engagement. Only 11 studies (27,5%) clearly distinguished data from interpretation. Finally, only 9 out of 40 studies (22,5%) displayed some form of reflexivity.

### Findings on accountability strategies

We found that that five areas of SRHR were discussed: maternal, neonatal and child health services, HIV services, gender-based violence, LGBT access and access to reproductive health care in general (Table 4).

**Table 4:** Overview of accountability studies per SRHR area, country, scale and its potential beneficiaries.

Accountability strategy per SRHR area	Country	Scale	Beneficiaries	Article
**Maternal, Neonatal and Child Health**				
National Guidelines on attention to women in labour and in delivery	Dominican Republic	National and sub-national	Pregnant women and women in labour in health facilities	Freedman 2003 [28]
Creation of national Nigeria Independent Accountability Mechanism	Nigeria	National	Not explicitly mentioned	Garba & Bandali 2014 [29]
Introduction policy on confidential inquiry	Nigeria	National	Not explicitly mentioned	Hussein & Okonufua 2012 [31]
Development civil registration and vital statistics (CRVS) and Maternal Death Surveillance and Response (MDSR) systems and audits	Low and Middle Income	National and sub-national	Pregnant women and neonates	Mathai et al. 2015 [30], Scott and Danel 2016 [32]
Development of pregnancy surveillance and registry system	India and Bangladesh	Sub-national	Pregnant women	Labrique et al. 2012 [34]
Quality improvement through introduction local perinatal mortality audit tool	South Africa and Bangladesh	Sub-national (health facility level)	Neonates	Pattison et al. 2009 [16]
Examination of social and institutional conditions of hospital setting within context of near-miss intervention	Benin	Sub-national (health facility level)	Women who had obstetric emergencies	Behague et al. 2008 [41]
Assessment of satisfaction of care through a questionnaire based on 7 categories of disrespect and abuse	Ethiopia	Sub-national (health facility level)	Women who had given birth vaginally	Asefah & Bekele 2015 [17]
Exploration of existing social accountability practices related to maternal health	Democratic Republic of Congo	Sub-national (district)	Women	Mafuta et al 2015 [22]
Training providers in respectful maternity care	Burkina Faso	Sub-national	Pregnant women and women in labour in health facilities	Ouédraogo et al 2014 [33]
Quality improvement of facility-based maternal and child health care through direct observation of provider practices	Ethiopia, Kenya, Madagascar, Rwanda, Tanzania	Sub-national (health facility level)	Pregnant women, women in labour and children in health facilities	Rosen et al. 2015 [18]
Introduction of MNCH score cards and stakeholder meetings	Ghana	Sub-national (district and region)	Pregnant women, communities	Blake et al. 2016 [38]
Introduction of community-based scorecards, dashboards, confidential enquiry and maternal death audits	Ethiopia, Malawi, Tanzania, Nigeria and Sierra Leone	National and sub-national	Pregnant women, communities	Hulton et al. 2014 [21]
Introduction of community monitoring for maternal health by NGOs	India	Sub-national (decentralized state level)	Disenfranchised women	Papp et al. 2013 [19]
NGO led strategic litigation for violation of Economic and Social Rights (ESR), case of maternal death	India	Sub-national (decentralized state level)	Poor women from lower caste communities	Kaur 2012 [43]
**HIV**				
Use of the Nigerian Constitution to protect against mandatory premarital HIV testing	Nigeria	National	HIV + people, HIV+ women in particular	Durojaye & Balogun 2010 [44]
Introduction of Accountability for Reasonableness model in district priority setting for PMTCT programme	Tanzania	Sub-national (district)	PMTCT programme users	Shayo et al. 2013 [23]
Assessment of fairness priority setting within regional HIV/AIDS control programme	Indonesia	Sub-national (regional)	Communities	Tromp et al. 2015 [26]
Description of accountability mechanisms within context of scale up of HIV services	Zambia	Sub-national (health facility level)	HIV services users	Topp et al. 2015 [25]
Description of planning within the context of scaling up male circumcision	Rwanda	National	Men	McPherson et al. 2014 [24]
**Gender-based violence**				
Use of the Constitution to enforce protection against sexual violence	South Africa	National	Victims of sexual violence	Du Toit 2016 [55]
Implementation of the Prohibition of Child Marriage Act (2006)	India	Sub-national (decentralized state level)	Children / girls	Ghosh 2011 [35]
Implementation of national reparation policy for victims of sexual violence	Post-conflict Guatemala and Peru	National	Indigenous, rural, poor women	Duggan et al. 2008 [54]
Implementation of UN Resolution 1325 through micro-initiatives by NGOs	Post-conflict LMIC (Afghanistan, Haiti, Israel/Palestine, Kosovo, Mongolia, Nepal, Philippines, Sri Lanka	Sub-national	Women	Barrow 2009 [37]
Participatory action research on NGO truth telling exercise survivors sexual violence	Post-conflict Guatemala	Sub-national	Women survivors sexual violence	Crosby & Lykesy 2011 [51]
Description of accountability strategies for post-conflict sexual violence related to documentation, investigation and prosecution of sexual violence	Kenya, Liberia, Sierra Leone, Uganda	National and sub-nation (police and prosecution units)	Victims of sexual violence	Seelinger 2014 [36]
**LGBT access**				
(Lack of) Supreme Court protection of ESR	India	National	Not explicitly mentioned	Khaitan 2015 [45]
Litigation by NGOs to hold government accountable for ESR violations of disenfranchised groups	India, Uganda, Belize	National and sub-national (national and local courts)	Disenfranchised groups	McCrudden 2015 [42]
Strategic litigation by activist lawyers to ensure LGBT rights	Chile, India	National	LGBT	Miles 2015 [46]
Strategic litigation by conservative NGOs to suspend implementation national abortion guidelines and LGBT rights	Argentina	Sub-national (provincial courts)	Not explicitly mentioned	Penas De Fago et al. 2014 [40]
Use of contradicting policies by policymakers to ensure support for their political agenda	Ecuador	National	Not Applicable	Lind & Keating 2015 [52]
**Reproductive health care in general**				
Legal case using the Constitution to hold non-state actors accountable for ESR violations	South Africa	National	People living in South Africa	Nolan 2014 [49]
Legal case using Minimum Core Approach within Constitution to protect ESR rights of marginalized groups and provide them with minimum essential levels of services	Kenya, South Africa, Colombia	National	Disenfranchised groups	Orago 2015 [50]
Legal cases using of Section 26 and 27 of the South African Constitution to ensure access to RH care	South Africa	National	Poor, disenfranchised groups	Bendana & Chopra 2013 [27]
Legal case using Constitution for ESR protection	Malawi	National	Disenfranchised groups	Chirwa 2005 [47]
The implementation of the protection of ESR under the Somaliland Constitution and the implementation of the national gender policy	Somaliland	National	Disenfranchised women	Bendana & Chopra 2013 [27]
Exploration of personal accountability child bearing practices against religious background and state development discourse	Morocco	Individual	Not Applicable	Rinker 2015 [53]
Examination of the range of accountability strategies in service accountability for reproductive health	India, Brazil, Bolivia, Bangladesh	National and sub-national	Marginalised groups, communities	George 2003 [39]
Litigation on the failure of providing regulation for the determination of parenthood (surrogacy mothers)	China	National and sub-national	Surrogate mothers	Ding 2015 [20]

### What are the main types of accountability strategies in SRHR?

In the 40 studies reviewed, we identified three main groupings of accountability strategies: performance accountability, social or ‘community’ accountability and legal accountability. Performance accountability mainly refers to the internal systems that governments hold service providers and health systems to account (see for instance maternal death surveillance and response (MDSR), VRSC, surveillance, etc.), while social accountability is about citizens holding service providers to account. Articles on both of these types predominantly focused on improving the quality of maternal, neonatal and child health care, and increasing coverage and service utilization.

Fifteen of the publications deal with *performance accountability* and they increase in number from 2012 onwards [16, 21, 24, 25, 29–35, 56]. As mentioned earlier, these articles related to “internal accountability” strategies in relation to service, managerial, administrative or programmatic issues. Specific accountability instruments included patient or death registration and surveillance systems, as well as staff performance review and disciplinary measures. Several articles stressed the need to guard against accountability measures being cast purely as punitive, and they call for a constructive framing of accountability as a way to improve service delivery [39, 56].

*Social or “community” accountability* is examined by nine articles. These studies sought to bolster the capacity of communities to demand improved service delivery and provider responsiveness through raising community awareness and voice [21–23, 26, 38–39, 41, 48, 51, 57]. We included the Accountability for Reasonableness studies [22, 25] in this category as they assess community involvement in priority-setting and democratic deliberative spaces through participatory tools and processes. We also included the ethnography of obstetric patients in Benin, as it reveals the factors that hold some patients back from demanding social accountability, as well as the nuanced ways in which others are able to negotiate with providers [41]. Among the specific instruments examined in these studies, are stakeholder meetings, public hearings, and the use of community scorecards and dashboards. More formalized mechanisms, such as village health or health watch committees, citizen charters or efforts to implement right to information legislation, also addressed sexual and reproductive health and rights.

The final thirteen articles related to *legal accountability* for SRHR (see for instance 27, 36, 40, 43–48, 50–51, 54–55). Broadly speaking, legal accountability is about holding the government accountable to wronged citizens and communities. These studies include investigations of accountability achieved through national legal systems, i.e. strategic or public litigation and tribunals. We can distinguish two sub-themes: one is related to accountability for human rights violations and the second is accountability for upholding constitutional rights. The former considers the violations and (lack of) protection of sexual and reproductive health and rights under a national legal and policy framework. These studies interpret the State’s role as duty-bearer to implement national and sub-national accountability strategies to protect citizen’s economic, social and cultural rights. These tended to be examples of NGOs, both progressive and conservative ones, use of strategic litigation or public interest litigation as an instrument to enforce accountability for infringements of human rights, for example in Chile [46], Argentina [40], China [20], Harayana state in India [43, 45]. In the second sub-theme, accountability for upholding constitutional rights, there was a particular focus on the protection of specific economic, social and cultural rights as outlined in the constitution, namely in South Africa, Nigeria, Malawi, Somaliland and Kenya [27, 44, 47–49, 55].

Furthermore, the studies related to legal accountability detailed how national policies and national legal systems increasingly play a role in delivering accountability [36]. A number of studies revolve around decision-making processes and the implementation of laws, policies, programmes and guidelines [23–25, 26, 37, 54, 55]. Another set of studies focus on civil society organisations, preparing or bringing cases on the violations of sexual and reproductive health and rights before court [35, 40, 42–43, 46, 51]. Finally, a group of studies focuses on the role of the courts and the possibilities within the respective countries’ constitutions to protect access to reproductive health and LGBT access [27, 44–45, 47–50, 55].

### What are the reported contexts for SRHR accountability to succeed?

Several of articles identified particular contextual conditions associated with successfully undertaking accountability for SRHR at national and sub-national level. Table 5 categorizes them in terms of broad social structures, governance factors, and core features of the health system. However, few of these contextual descriptions were detailed in nature. Often, context was presented in the background section of the article, without explicit analysis of its contribution to the observed outcomes or linkage to accountability mechanisms. For example, we did not find articles that specifically mentioned the media or the extent of privatization of health services as contextual factors influencing accountability for sexual and reproductive health and rights.

**Table 5:** Contextual conditions for successful SRHR accountability.

Reported context conditions	Studies
**Broad social structure**	
Societal awareness (e.g. no fear of stigma for victims of SRHR violations)	Seelinger, 2014 [36], Duggan et al. 2008, [54], George 2003 [39]
Active civil society and civic culture (advocating for the implementation of SRHR through strategic litigation, amongst other strategies)	Chirwa, 2005 [47], George 2003 [39]
Trust in the legal system and the institutions	Bendana & Chopra, 2013 [27], Seelinger, 2014 [36]
**Governance context (overall political and legal framework)**	
Democratic space (civil society action is possible)	Miles, 2015 [46], Lind & Keating, 2013 [52]
Recognition of the rule of law, reduced impunity (freedom from reprisal when victims report violations)	Bendana & Chopra, 2013 [27], Seelinger, 2014 [36], Duggan et al., 2008 [54]
Independent judiciary knowledgeable about human rights and SRHR	Khaitan, 2015 [45], Kaur, 2012 [43], Seelinger, 2014 [36]
Adapted legal and policy framework	Scott & Danel, 2016 [32]
**Health system context**	
Community participation in the health system	Scott & Danel, 2016 [32], George 2003 [39]
Adequately resourced health system (timely budget allocation, adequate human resources)	Scott & Danel, 2016 [32]
Motivated health providers and no blame culture in health facilities	Scott & Danel, 2016 [32], Asefa & Bekele, 2015 [17]
Robust Health Management and Information System	Mathai et al., 2015 [30]
Sound management of the local health system and the health facility, leadership	Freedman, 2003 [28], Blake et al., 2016 [38]

### What are the reported outcomes?

The studies reviewed reported several types of outcomes (See S6 Table). Not surprisingly, few studies were able to document health outcomes due to their study designs. Authors more frequently focused on intermediary outcomes, such as community or health care user empowerment, provider behaviour, broader health systems or changes in legislation, policy or guidelines changes.

Hussein and Okonufua [31] summarize the effects of accountability interventions in maternal health on provider practices. They reported mixed changes in the professional practice of health workers, with better outcomes in multifaceted interventions compared to those focused solely on audit and feedback. Hussein and Okonufua conclude that much uncertainty exists on the effectiveness of audits, with some studies showing no evidence and others revealing inconclusive findings. Concerning changes at the level of national and sub-national levels, both Mathai et al. [30] and Scott and Danel [32] reported an increase in the implementation of maternal death surveillance and response committees, national level confidential enquiry or maternal death review committees. Both Hussein and Okonufua [31] and Pattinson et al. [16] reported on the cost effectiveness of the implementation of maternal death audits in resource-constrained settings. The most substantial cost these studies cited related to data collection and analysis.

Topp et al. [25] and Papp et al. [19] reported positive changes in capabilities of disenfranchised groups. Topp et al. found a positive effect on the empowerment of people living with HIV, while Papp. et al. noted that women’s capability to demand accountability improved. Other reported outcomes found in the review relate to changes in the content of policies or in the pace or progress of implementation. For example, gender laws in Nepal and Sri Lanka were modified [37] as a result of civil society demands, and a court in the Indian state of Madhya Pradesh ordered the immediate implementation of maternal death audits [43].

Three studies reported unintended effects. Topp et al. [25] found that the attention given to donor-driven HIV services scaling up in Zambia, which was accompanied by a number of accountability strategies, had a negative impact on quality of care for patients in need of other health services. Lind & Keating [52] found that political capture of LGBT issues masked the lack of progress in other critical ESR obligations. Two articles outlined how religiously affiliated NGOs use strategic litigation to repeal implementation of SRHR laws and related policies [40, 42].

We also assessed whether the studies reported any outcomes related to increased equity. Few studies reported evidence on the equity effects of accountability strategies, though several commented on their potential to influence equity positively. For example, accountability strategies involving civil society organization’s use of strategic litigation and constitutional accountability point to their potential to enforce access for disenfranchised groups [49] and their long-term potential to contribute to the transformation of power dynamics [27, 35, 43–44, 47–48, 50–51, 54]. George [39] notes that despite the transformational intent of participatory approaches, social inclusion and legitimate representation of marginalized groups are not achieved automatically.

## Discussion

Our review confirms the rising importance of accountability initiatives in SRHR as signalled by the increase in publications in 2014 and 2015. While the bulk of the articles are drawn from public health, a significant number of articles reflect legal perspectives, as well as contributions from other social science disciplines. The public health studies were largely qualitative case studies, with very few ethnographic, action research or critical studies contributions. The quality of the studies was hard to assess given the diverse disciplinary background of the articles.

The review classed the accountability articles into three main strategies: performance, social and legal accountability. While the majority of articles on performance and social accountability strategies focused on improving service delivery for maternal, neonatal and child health, legal and policy activism aimed at addressing accountability for HIV, GBV and LGBT concerns.

The review confirms the emerging analytic paradigm that treats accountability interventions as situated within complex accountability ecosystems comprised of multiple actors and institutions with a range of roles, responsibilities, interactions, and incentives. These ecosystems operate at multiple levels, from the transnational to the local.

These accountability strategies change the terms of engagement among the actors involved. Our review highlights that accountability is not a ‘one size fits all’ formulation where a set of prescribed tools can be transferred from one setting to another with an expectation of achieving similar outcomes. Rather, the success of accountability strategies is influenced by context-specific factors including power relations, socio-cultural dynamics, and the ability of community to negotiate accountability. Thus, our review’s finding align with analyses of accountability strategies and interventions beyond SRHR [57].

The recommendations made by the Commission on Information and Accountability for Women’s and Children’s Health in 2011 [58] coincide with a rise in publications on performance accountability after 2013. The legal studies reveal a clear use of global legal norms in litigation at constitutional courts or as part of special mechanisms or tribunals, citing, for example, the 1976 International Covenant on Economic, Social and Cultural Rights Economic and Social Rights and UN Security Council Resolution 1325. These studies offer suggestive evidence that global normative frameworks are influencing national laws and policies. In these instances, global norms and standards provide transnational legitimacy for reformers seeking to pursue national accountability efforts.

In terms of impacts on health, the bulk of articles focused on MNCH, though several articles documented accountability experiences related to HIV, gender-based violence, LGBT or reproductive health. No published articles were found related to safe abortion, reproductive cancers or family planning, despite the active social movements and the role of litigation supporting policy and programming in those areas.

In terms of specific populations, articles did reflect the experience of reproductive age women, HIV affected populations and LGBT communities. While several articles reported accountability measures for marginalized communities, the specific experiences of adolescents and sex workers were not captured by the studies reviewed. While several articles listed marginalized communities as their main concern, authors tended not to address the equity effects of the accountability strategies being assessed. No publications examined how the accountability strategies addressed structural inequalities and benefits distribution across populations.

Finally, we note certain gaps in the published literature with regards to other types of accountability strategies beyond those in the three categories our review examined. The studies reviewed paid little attention to parliaments, a traditional institution for public accountability in democratic governance models; to national human rights bodies; or to the effects of elections or protest actions. We did not find studies discussing parliamentary committee works such as budget committees, nor parliamentary hearings on sexual and reproductive health and rights. Also absent were references to ombudsman and whistle-blower strategies and administrative sanctioning procedures as accountability instruments. Financial accountability, and related tools such as participatory budgeting, are also missing in the published literature for sexual and reproductive health and rights.

### Strengths and limitations

One of the strengths of this review is that it gathered articles from diverse disciplines. This has broadened our understanding of accountability ecosystems in SRHR, and particularly of how they change the terms of engagement between the actors involved. A second strength is that the review covered not only specific interventions but also approaches such as civil society action and litigation.

Arguably, this review only represents a sliver of what is happening on the ground as it was limited to the peer-reviewed literature. It therefore necessarily reflects the academic evidence base on accountability in health or other sectors. Much of the evidence related to civil society action in sexual and reproductive health and rights has not been published in peer-review journals. A wider review of accountability in the grey literature would be necessary to address the noted evidence gaps. Nevertheless, limitations will likely remain as documentation of actions by practitioners such as activist civil society organisations is often neither their priority especially given the resource constraints they often face.

Another limitation is related to language: only LexisNexis Academic allowed for selection other languages than English. Finally, there may be some bias in the selection of studies retained in the review, as only 3 sets of 20 abstracts, drawn from the papers selected by each database were checked for adherence to the inclusion/exclusion by a second researcher. We acknowledge this constituted a small sample.

## Conclusion

As we note above, our review highlighted the importance of viewing accountability as located within accountability ecosystems. However, the current state of research provides little insight on how SRHR accountability strategies work as part of an accountability ecosystem and under which conditions. This gap is not specific to studies of SRHR, but is a challenge to research on accountability across sectors. We welcome the increased focus on accountability across different dimensions of health, particularly in relation to sexual and reproductive health and rights. However, policymakers and practitioners are often under pressure to identify what appear to be simple solutions, which run the risk of reducing accountability interventions to tokenism or quick fixes. A more nuanced understanding of contextual factors and their impacts on different strategies and processes and the capability of individuals and communities to negotiate accountability lies at the heart of ensuring that accountability efforts affirm sexual and reproductive health and rights.

## Supporting information

S1 Table The search strategy. (DOCX)

S2 Table The search results. (DOCX)

S3 Table Data quality appraisal. (DOCX)

S4 Table The papers included in the review. (DOCX)

S5 Table Summary of reported outcomes by type. (DOCX)

S1 File PRISMA checklist. (DOC)
