# Correction: Broadening understanding of accountability ecosystems in sexual and reproductive health and rights: A systematic review

**DOI:** 10.1371/journal.pone.0200675

**Published:** 2018-07-10

**Authors:** Sara Van Belle, Vicky Boydell, Asha S. George, Derick W. Brinkerhoff, Rajat Khosla

The fourth author’s name is spelled incorrectly. The correct name is: Derick W. Brinkerhoff. The correct citation is: Van Belle S, Boydell V, George AS, Brinkerhoff DW, Khosla R (2018) Broadening understanding of accountability ecosystems in sexual and reproductive health and rights: A systematic review. PLoS ONE 13(5): e0196788. https://doi.org/10.1371/journal.pone.0196788
