# Supplementary material for: Broadening understanding of accountability ecosystems in sexual and reproductive health and rights: A systematic review
Source: PLoS One. 2018 May 31;13(5):e0196788. doi: 10.1371/journal.pone.0196788 (PMC5978882; doi:10.1371/journal.pone.0196788)
Supplement: S1 Table — (DOCX) [file pone.0196788.s001.docx]

**S1 - The search strategy**

| **LexisNexis Academic (1994-2016): UK/European journals and Asian, Brazilian and French language law journals: search for articles pertaining to LMIC** | | | |
| --- | --- | --- | --- |
| Accountab* and Sexual* and Reproductive In All Fields |  | UK/European journals | 116 |
| Accountab* and Sexual* and Reproductive In All Fields |  | Other | 22 |
| Accountability and LGBT In All Fields |  | UK/European journals | 51 |
| Accountability and LGBT In All Fields |  | Other | 0 |
| Accountability and Intersex in All Fields |  | UK/European | 7 |
| Accountability and Intersex in All Fields |  | Other | 1 |
| Sexual and Rights in TI, accountability in All Fields |  | UK/European | 1 |
| Sexual and Rights in TI, accountability in All Fields |  | Other | 0 |
| Reproductive and Rights in TI, accountability in All Fields |  | UK/European | 2 |
| Reproductive and Rights in TI, accountability in All Fields |  | Other | 0 |
| Accountability in TI, quality and care and sexual in All Fields |  | UK/European | 7 |
| Accountability in TI, quality and care and sexual in All Fields |  | Other | 0 |
| Accountability in TI, quality and care and reproductive in All Fields |  | UK/European | 0 |
| Accountability in TI, quality and care and reproductive in All Fields |  | Other | 0 |
| Accountability and acceptability and sexual in All Fields |  | UK/European | 72 |
| Accountability and acceptability and sexual in All Fields |  | Other | 1 |
| Accountability and acceptability and reproductive in All Fields |  | UK/European | 24 |
| Accountability and acceptability and reproductive in All Fields |  | Other | 0 |
| Accountability and accessibility and sexual in All Fields |  | UK/European | 58 |
| Accountability and accessibility and sexual in All Fields |  | Other | 0 |
| Accountability and accessibility and reproductive in All Fields |  | UK/European | 22 |
| Accountability and accessibility and reproductive in All Fields |  | Other | 0 |
| Accountability and availability in All Fields, Sexual in TI |  | UK/European | 4 |
| Accountability and availability in All Fields, Sexual in TI |  | Other | 0 |
| Accountability and participation in All Fields, Sexual in TI |  | UK/European | 13 |
| Accountability and participation in All Fields, Sexual in TI |  | Other | 0 |
| Accountability and participation in All Fields, Reproductive in TI |  | UK/European | 3 |
| Accountability and participation in All Fields, Reproductive in TI |  | Other | 0 |
| Accountability and Abortion, occurring at least 5 times |  | UK/European | 5 |
| Accountability and Abortion, occurring at least 5 times |  | Other | 0 |
| Accountability and Sterilization, occurring at least 5 times |  | UK/European | 1 |
| Accountability and Sterilization, occurring at least 5 times |  | Other | 0 |
| Accountability and Informed and Decision and Sexual, occurring at least 5 times |  | UK/European | 7 |
| Accountability and Informed and Decision and Sexual, occurring at least 5 times |  | Other | 0 |
| Accountability and Informed and Decision in All Fields and Reproductive, occurring at least 5 times |  | UK/European | 13 |
| Accountability and Informed and Decision in All Fields and Reproductive, occurring at least 5 times |  | Other | 0 |
| Accountability and Confidentiality and Sexual in All Fields |  | UK and European | 161 |
| Accountability and Confidentiality and Sexual in All Fields |  | Other | 3 |
| Accountability in All Fields and Privacy in title and Sexual in All Fields |  | UK and European | 8 |
| Accountability in All Fields and Privacy in title and Sexual in All Fields |  | Other | 0 |
| Accountability and Privacy and Reproductive in All Fields |  | UK and European | 45 |
| Accountability and Privacy and Reproductive in All Fields |  | Other | 0 |
| Accountability and Non-Discrimination and Reproductive In All Fields |  | UK and European | 59 |
| Accountability and Non-Discrimination and Reproductive In All Fields |  | Other | 0 |
| Accountability and Non-Discrimination In All Fields and Sexual in Title |  | UK and European | 3 |
| Accountability and Non-Discrimination In All Fields and Sexual in Title |  | Other | 0 |
| Accountability and Equity In All Fields and sexual in Title |  | UK and European | 5 |
| Accountability and Equity In All Fields and sexual in Title |  | Other | 0 |
| Accountability and Equity In All Fields and reproductive in Title |  | UK and European | 1 |
| Accountability and Equity In All Fields and reproductive in Title |  | Other | 0 |
| Accountability and Equality In All Fields and reproductive in TI |  | UK and European | 3 |
| Accountability and Equality In All Fields and reproductive in TI |  | Other | 0 |
| Accountability and Equality In All Fields and sexual in TI |  | UK and European | 11 |
| Accountability and Equality In All Fields and sexual in TI |  | Other | 0 |
| Accountability and Stigma In All Fields and sexual in TI |  | UK and European | 12 |
| Accountability and Stigma In All Fields and sexual in TI |  | Other | 0 |
| Accountability and Stigma In All Fields and reproductive in TI |  | UK and European | 3 |
| Accountability and Stigma In all Fields and reproductive in TI |  | Other | 0 |
