# Supplementary material for: Broadening understanding of accountability ecosystems in sexual and reproductive health and rights: A systematic review
Source: PLoS One. 2018 May 31;13(5):e0196788. doi: 10.1371/journal.pone.0196788 (PMC5978882; doi:10.1371/journal.pone.0196788)
Supplement: S2 Table — (DOCX) [file pone.0196788.s002.docx]

## S1 - The search results

| **Search terms and Boolean operators** | **Category** | **No. of records yielded from database** |
| --- | --- | --- |
| **Web of Science (1994-2016)** | |  |
| Accountability AND Sexual AND Reproductive AND Health in TI | In TI | 2 |
| Accountab* AND Sexual in TI | In TI | 12 |
| Accountab* AND Reproductive in TI | In TI | 6 |
| Answerab* AND Reproductive in TI | In TI | 0 |
| Answerab* AND Sexual | In TI | 0 |
| Enforc* AND sexual in TI | In Ti | 27 |
| Enforc* AND reproductive in TI | In TI | 5 |
| Parliament AND Reproductive | In TI | 4 |
| Parliament AND Sexual* | In TI | 3 |
| Ombudsman AND Reproductive | In TI | 0 |
| Ombudsman AND Sexual* | In TI | 3 |
| Professional* AND Association AND Sexual* | In TI | 1 |
| Professional* AND Association AND Reproductive | In TI | 4 |
| Accountab* AND Professional AND Sexual* | In TI | 0 |
| Accountab* AND Professional AND Reproductive | In TI | 0 |
| Committee AND Reproductive AND Accountab* | In TI | 0 |
| Committee AND Sexual AND Accountab* | In TI | 0 |
| Commission AND Reproductive AND Accountab* | In TI | 0 |
| Commission AND Sexual AND Accountab* | In TI | 0 |
| Commission AND Sexual* AND Admin* | In TI | 0 |
| Commission AND Reproductive AND Admin* | In TI | 0 |
| Commission AND Sexual AND Justice | In TI | 1 |
| Commission AND Reproductive AND Justice | In TI | 0 |
| Information AND Reproductive AND Right* | In TI | 7 |
| Information AND sexual AND right* | In TI | 5 |
| Information AND reproductive AND accountab* | In TI | 0 |
| Information AND Sexual AND Accountab* | In TI | 0 |
| Reproductive AND Consumer AND Forum | In TI | 0 |
| Sexual* AND Consumer AND Forum | In TI | 0 |
| Reproductive AND Commissioner | In TI | 0 |
| Sexual* AND Commissioner | In TI | 3 |
| Reproductive AND Citizen AND Score* | In TI | 0 |
| Sexual AND Citizen AND Score* | In TI | 0 |
| Sexual* AND Citizen* | In TI | 31 |
| Reproductive AND Citizen* | In TI | 6 |
| Reproductive AND Constitution | In TI | 14 |
| Sexual* AND Constitution | In TI | 16 |
| Sexual* AND Health AND Summit | In TI | 5 |
| Reproductive AND Health AND Summit | In TI | 5 |
| Reproductive AND Health AND Sector AND Review | In TI | 0 |
| Sexual* AND Health AND Sector AND Review | In TI | 0 |
| Sexual* AND Health AND Council | In TI | 2 |
| Reproductive AND Health AND Council | In TI | 0 |
| Reproductive AND Criminal AND Investig* | In TI | AND |
| Sexual AND Criminal AND Investig* | In TI | 7 |
| Reproductive AND Board | In TI | 12 |
| Sexual* AND Board | In TI | 31 |
| Sexual* AND Accreditation | In TI | 8 |
| Reproductive AND Accreditation | In TI | 6 |
| Reproductive AND Patient AND Groups | In TI | 2 |
| Sexual* AND Patient AND Groups | In TI | 36 |
| Reproductive AND User AND Groups | In TI | 3 |
| Sexual* AND User AND Groups | In TI | 7 |
| Sexual* AND Charter | In TI | 7 |
| Reproductive AND Charter | In TI | 1 |
| Sexual AND Audit AND Accountab* | In TI | 0 |
| Reproductive AND Audit | In TI | 6 |
| Sexual AND Grievance | In TI | 6 |
| Reproductive AND Grievance | In TI | 0 |
| Sexual* AND Grievance | In TI | 6 |
| Reproductive AND Grievance | In TI | 0 |
| Sexual AND Redress | In TI | 11 |
| Reproductive AND Redress | In TI | 0 |
| Sexual AND Remed* | In TI | 6 |
| Reproductive AND Remed* | In TI | 21 |
| Sexual* AND Community AND Accountab* | In TI | 6 |
| Reproductive AND Community AND Accountab* | In TI | 1 |
| Sexual* AND Monitoring AND Accountab* | In TI | 0 |
| Reproductive AND Monitoring AND Accountab* | In TI | 0 |
| Sexual* AND Enforc* | In TI | 36 |
| Reproductive AND Enforc* | In TI | 5 |
| Sexual AND Regulat* AND Accountab* | In TI | 0 |
| Reproductive AND Regulat* AND Accountab* | In TI | 0 |
| Sexual* AND Feedback AND Accountab* | In TI | 0 |
| Reproductive AND Feedback AND Accountab* | In TI | 0 |
| Reproductive AND Complaint | In TI | 7 |
| Sexual* AND Complaint | In TI | 73 |
| Sexual* AND Protest | In TI | 9 |
| Reproductive AND Protest | In TI | 1 |
| Sexual* AND Litig* | In TI | 24 |
| Reproductive AND Litig* | In TI | 9 |
| **Pubmed (1994-2016)** | | |
| Accountab* AND Sexual* | In TI | 6 |
| Accountab* AND Reproductive | In TI | 5 |
| Answer* AND Sexual* | In TI | 32 |
| Answer* AND Reproductive | In TI | 12 |
| Sexual* AND Enforc* | In TI | 11 |
| Reproductive AND Enforc* | In TI | 3 |
| Accountab* AND Gender | In TI | 3 |
| Accountab* AND Violence | In TI | 8 |
| Accountab* AND Maternal | In TI | 11 |
| Accountab* AND Childbirth | In TI | 1 |
| Accountab* AND Pregnanc* | In TI | 1 |
| Accountab* AND Obstetric | In TI | 2 |
| Accountab* AND Teenage | In TI | 1 |
| Accountab* AND Abortion | In TI | 2 |
| Accountab* AND Adolescent | In TI | 3 |
| Accountab* AND HIV | In TI | 20 |
| Accountab* AND Referral | In TI | 0 |
| Accountab* AND Antenatal | In TI | 0 |
| Accountab* AND Contracept* | In TI | 0 |
| Accountab* AND Family AND Planning | In TI | 0 |
| Accountab* AND Fertility | In TI | 3 |
| Accountab* AND PMTCT | In TI | 0 |
| Accountab* AND perinatal | In TI | 5 |
| Accountab* AND fistula | In TI | 1 |
| Accountab* AND Abuse | In TI | 20 |
| Accountab* AND Child | In TI | 70 |
| Accountab* AND Respectful | In TI | 0 |
| Respectful AND Childbirth | In TI | 2 |
| Respectful AND Care | In TI | 20 |
| Respect* AND Pregnanc* | In TI | 28 |
| Respect* AND Reproduct* | In TI | 33 |
| Respect* AND Sexual* | In TI | 40 |
