# Supplementary material for: Broadening understanding of accountability ecosystems in sexual and reproductive health and rights: A systematic review
Source: PLoS One. 2018 May 31;13(5):e0196788. doi: 10.1371/journal.pone.0196788 (PMC5978882; doi:10.1371/journal.pone.0196788)
Supplement: S3 Table — (DOCX) [file pone.0196788.s003.docx]

**S4 - Data quality appraisal: Mays and Pope criteria (2000) and quality appraisal table**

- Did the researcher(s) create an adequate account of the methods and data analysis process?  This account should be able to stand independently such that another trained researcher could analyze the data in the same way and reach the same conclusions ([audit trail](http://www.qualres.org/HomeAudi-3700.html))? (column “audit” in table below)
- Is the sampling process described adequately?  Is there a coherent rationale presented for the sampling strategy, and is the investigator identifying participants or examining circumstances that are relevant to the research question? (column “sampling” in table below)
- Validity in qualitative research can be attained by appropriate use of some of the following strategies: triangulation, deviant case analysis, member checking (column “validity” in table below)
- How well does the research account explain why people behave as they do? (column “explanatory power” in table below)
- How comprehendible is the account to someone in the setting? (column “insider” in table below)
- How does the report correspond with and advance current knowledge? (column “knowledge” in table below)
- Does the report have sufficient detail for an outsider to function in the setting that the investigator studied? (column “detail” in table below)
- Was there evidence of [prolonged engagement](http://www.qualres.org/HomeProl-3690.html) in the field? (column “field” in table below)
- Can the reader distinguish data from interpretation? (column “data vs interpretation” in table below)
- -Did the research reflect on how the methods chosen and used might have influenced or shaped the data collected and the interpretations made ([reflexivity](http://www.qualres.org/HomeRefl-3703.html))? (column “reflexivity” in table below)

**Legend of score the criteria**

Yes / no”

+: low

++ : moderate / average,

+++:high/strong

NA: criteria not applicable

|  | **Audit** | **Sampling** | **Validity** | **Explan.power** | **Insider** | **Knowledge** | **Detail** | **Field** | **Data vs. interpretation** | **Reflexivity** |
| --- | --- | --- | --- | --- | --- | --- | --- | --- | --- | --- |
| **1.** | **yes** | **yes** | **NA** | **+++** | **NA** | **+++** | **+++** | **NA** | **yes** | **NA** |
| **2.** | **yes** | **yes** | **no** | **+** | **++** | **++** | **+** | **no** | **no** | **no** |
| **3.** | **yes** | **no** | **NA** | **+++** | **+** | **+** | **+** | **no** | **no** | **yes** |
| **4.** | **no** | **no** | **no** | **+++** | **++** | **+++** | **++** | **yes** | **no** | **yes** |
| **5.** | **yes** | **yes** | **yes** | **+++** | **+++** | **+++** | **+++** | **yes** | **yes** | **no** |
| **6.** | **no** | **NA** | **no** | **++** | **+** | **++** | **+** | **yes** | **no** | **no** |
| **7.** | **yes** | **yes** | **NA** | **+++** | **++** | **++** | **++** | **yes** | **no** | **no** |
| **8.** | **no** | **NA** | **no** | **+** | **+++** | **+** | **+** | **yes** | **no** | **no** |
| **9.** | **no** | **NA** | **no** | **+** | **+** | **++** | **+** | **yes** | **no** | **no** |
| **10.** | **no** | **NA** | **no** | **+** | **++** | **+** | **+** | **no** | **no** | **no** |
| **11.** | **no** | **no** | **no** | **+++** | **++** | **++** | **+** | **no** | **no** | **no** |
| **12.** | **no** | **no** | **no** | **++** | **++** | **+** | **+** | **no** | **no** | **no** |
| **13.** | **no** | **NA** | **yes** | **+++** | **+++** | **+++** | **+** | **no** | **no** | **no** |
| **14.** | **no** | **no** | **no** | **+++** | **+** | **+++** | **++** | **yes** | **no** | **no** |
| **15.** | **no** | **NA** | **yes** | **+++** | **++** | **+++** | **++** | **yes** | **no** | **no** |
| **16.** | **yes** | **yes** | **yes** | **+** | **++** | **++** | **+** | **yes** | **yes** | **no** |
| **17.** | **yes** | **NA** | **no** | **++** | **+** | **++** | **+** | **yes** | **no** | **yes** |
| **18.** | **no** | **no** | **no** | **++** | **+** | **+++** | **+** | **NA** | **no** | **no** |
| **19.** | **no** | **no** | **NA** | **+** | **+++** | **+++** | **++** | **yes** | **no** | **no** |
| **20.** | **no** | **NA** | **no** | **+** | **+** | **+** | **+** | **NA** | **no** | **no** |
| **21.** | **no** | **no** | **no** | **++** | **++** | **++** | **+** | **no** | **no** | **no** |
| **22.** | **no** | **NA** | **no** | **+++** | **+** | **+++** | **+** | **no** | **no** | **yes** |
| **23.** | **yes** | **yes** | **yes** | **+++** | **++** | **++** | **++** | **no** | **yes** | **yes** |
| **24.** | **no** | **NA** | **no** | **+** | **++** | **++** | **+** | **NA** | **no** | **no** |
| **25.** | **no** | **NA** | **no** | **++** | **++** | **+** | **+** | **no** | **no** | **no** |
| **26.** | **no** | **no** | **no** | **+** | **+** | **+** | **+** | **no** | **yes** | **no** |
| **27.** | **yes** | **yes** | **yes** | **++** | **++** | **+++** | **++** | **yes** | **no** | **no** |
| **28.** | **no** | **NA** | **yes** | **++** | **+** | **+** | **+** | **NA** | **no** | **no** |
| **29.** | **no** | **NA** | **no** | **+** | **+** | **++** | **+** | **NA** | **no** | **no** |
| **30.** | **yes** | **yes** | **yes** | **++** | **++** | **+** | **++** | **yes** | **yes** | **yes** |
| **31.** | **yes** | **yes** | **yes** | **+** | **++** | **+** | **+** | **no** | **no** | **no** |
| **32.** | **yes** | **NA** | **NA** | **+++** | **+** | **+++** | **+** | **NA** | **yes** | **NA** |
| **33.** | **no** | **no** | **no** | **+++** | **++** | **+++** | **+** | **no** | **no** | **no** |
| **34.** | **yes** | **yes** | **yes** | **+** | **+** | **+** | **+** | **yes** | **no** | **yes** |
| **35.** | **yes** | **yes** | **yes** | **+++** | **++** | **+++** | **+++** | **yes** | **yes** | **yes** |
| **36.** | **no** | **no** | **no** | **+** | **+++** | **+** | **+++** | **no** | **no** | **no** |
| **37.** | **yes** | **yes** | **yes** | **++** | **++** | **++** | **+** | **yes** | **no** | **no** |
| **38.** | **yes** | **yes** | **yes** | **++** | **++** | **++** | **+++** | **yes** | **yes** | **no** |
| **39.** | **yes** | **yes** | **yes** | **++** | **++** | **++** | **++** | **yes** | **yes** | **no** |
| **40.** | **yes** | **yes** | **yes** | **++** | **+++** | **++** | **+** | **no** | **yes** | **yes** |
