# Supplementary material for: Broadening understanding of accountability ecosystems in sexual and reproductive health and rights: A systematic review
Source: PLoS One. 2018 May 31;13(5):e0196788. doi: 10.1371/journal.pone.0196788 (PMC5978882; doi:10.1371/journal.pone.0196788)
Supplement: S4 Table — (DOCX) [file pone.0196788.s004.docx]

## S3 – The papers included in the review

**1. Asefah**, A., Bekele, D. (2015) Status of respectful and non-abusive care during facility-based childbirth in a hospital and health centers in Addis Ababa, Ethiopia in Reproductive Health, 12:33.

**2. Barrow**, A., (2009) (It’s) like a rubber band. Assessing UNSCR 1325 as a gender mainstreaming process in International Law in Context, Vol.5, no.1: 51-68.

**3. Béhague**, D.P., Kanhonou, LG., Filippi, V., Lègonou, S., Ronsmans, C. (2008) Pierre Bourdieu and transformative agency: a study of how patients in Benin negotiate blame and accountability in the context of severe obstetric events in Sociology of Health & Illness, Vol.30, no.4: 489-510.

**4. Bendana**, A., Chopra, T. (2013) Women’s Rights, State-Centric Rule of Law, and Legal Pluralism in Somaliland in Hague Journal on the Rule of Law, Vol.55: 44-73.

**5. Blake**, C., Annorbah-Sarpei, N.A., Bailey, C., Ismaila, Y., Deganus, S., Bosomprah, S., Galli, F., Clark, S. (2016) Scorecards and social accountability for improved maternal and newborn health services: A pilot in the Ashanti and Volta Regions of Ghana in International Journal of Gynecology and Obstetrics, <http://dx.doi.org/10.1016/ijgo.2016.10.004>.

**6. Chirwa**, D.M., (2005) A Full Loaf is Better than Half: The Constitutional Protection of Economic, Social and Cultural Rights in Journal of African Law, Vol.49, no.2: 204-241.

**7. Crosby**, A., Lykesy, M.B. (2011) Mayan Women Survivors Speak: The Gendered Relations of Truth Telling in Postwar Guatemala in International Journal of Transitional Justice, Vol.5, no.3: 456-476.

**8. Davis**, D.M., (2008) Socioeconomic rights: do they deliver the goods? In International Journal of Constitutional Law, Vol.6, no.4: 687-711.

**9. Ding**, C. (2015) Surrogacy Litigation in China and beyond in Journal of the Law and Biosciences, Vol.2, no.1: 33-35.

**10. Du Toit**, L. (2016) The South African Constitution as Memory and Promise: An Exploration of Its Implications for Sexual Violence, Politikon (South African Journal of Political Studies), Vol.43, no.1: 31-51.

**11. Duggan**, C., Paz y Paz Bailey C., Guillerot, J. (2008) Reparations for Sexual and Reproductive Violence: Prospects for Achieving Gender and Justice in Guatemala and Peru in International Journal of Transitional Justice, Vol.2, no.2: 192-213.

**12. Durojaye**, E., Balogun, V. (2010) Human Rights Implications of Mandatory Premarital HIV Testing in International Journal of Law, Policy and the Family, vol.24, no.2: 245-265.

**13. Freedman**, L.P., (2003) Human rights, constructive accountability and maternal mortality in the Dominican Republic: a commentary in International Journal of Gynecology and Obstetrics, Vol.83, no.2: 111-114.

**14. Garba**, A., Bandali, S. (2014) The Nigeria Independent Accountability Mechanism for maternal, newborn, and child health in International Journal of Gynecology and Obsterics, Vol.127, no.1: 113-116.

**15. George**, A. (2003) Using Accountability to Improve Reproductive Health Care in Reproductive Health Matters, Vol.11, no.21: 161-170.

**16. Ghosh**, B., (2011) Child Marriage, Society and the Law: A Study in a Rural Context in West Bengal, India in International Journal of Law, Policy and the Family, Vol.25, no.2: 199-219.

**17. Hulton**, L., Matthews, Z., Martin-Hilber, A., Adanu R., Ferla, C., Getachew, A., Makwenda, C., Segun, B., Yilla, M. (2014) Using evidence to drive action: A “revolution in accountability” to implement quality care for better maternal and newborn health in Africa in International Journal of Gynecology and Obstetrics, Vol.127: 96-101.

**18. Hussein**, J., Okonufua, F. (2012) Time for Action: Audit, Accountability and Confidential Enquiries into Maternal Deaths in Nigeria in African Journal of Reproductive Health, March, 16(1): 9-14.

**19. Kaur**, J., (2012) The role of litigation in ensuring women’s reproductive rights: an analysis of the Shanti Devi judgement in India. In Reproductive Health Matters, Vol. 20, no.39: 21-30.

**20. Khaitan**, T. (2015) Koushal v Naz: Judges Vote to Recriminalise Homosexuality in Modern Law Review, Vol.78, no.4: 672-680.

**21. Labrique**, A.B., Pereira, S., Christian, P., Murthy, N., Bartlett, L., Mehl, G. (2012) Pregnancy registration systems can enhance health systems, increase accountability and reduce mortality in Reproductive Health Matters, Vol.20, no.39: 113-117.

**22. Lind,** A., Keating, C. (2013) Navigating the Left Turn in International Feminist Journal of Politics, Vol.15, no.4: 515-533.

**23. Mafuta**, E.M., Dieleman, M.A., Hogema, L.M., Khomba, P.A., Zioko, F.M., Kayembe, P.K., De Cock Buning, T., Mambu, T.N.M. (2015) Social accountability for maternal health services in Muanda and Bolenge Health Zones, Democratic Republic of Congo: a situation analysis in BMC Health Services Research, Vol.15: 514.

**24. Mathai**, M., Dilip, T.R., Jawad, I., Yoshida, S. (2015) Strenghtening accountability to end preventable maternal deaths. In International Journal of Gynecology and Obstetrics, Vol.131: S3-S5.

**25. McCrudden**, C. (2015) Transnational Culture Wars in International Journal of Constitutional Law, Vol.13, no.2: 434-462.

**26**. **McPherson**, D.B., Balisanga, H.N., Mbabazi, J.K. (2014) Bridging the accountability divide: male circumcision planning in Rwanda as a case study in how to merge divergent operational planning approaches in Health Policy and Planning, Vol.29: 883-892.

**27. Miles**, P. (2015) Brokering Sexual Orientation and Gender Identity: Chilean Lawyers and Public Interest Litigation Strategies In Bulletin of Latin American Research, Vol.34, no.4: 435-450.

**28. Nolan**, A. (2014) Holding non-state actors to account for constitutional economic and social rights violations: Experiences and lessons from South Africa and Ireland in International Journal of Constitutional Law, vol.12, no.1: 61-93.

**29. Orago**, N.W. (2015) The Place of the “Minimum Core Approach” in the Realisation of the Entrenched Socio-Economic Rights in the 2010 Kenyan Constitution in Journal of African Law, Vol.59, no.2: 237-270.

**30. Ouédraogo**, A., Kiemtoré, S., Zamané, H., Bonané, B.T., Akotionga, M., Lankoande J. (2014) Respectful maternity care in three health facilities in Burkina Faso: The experience of the Society of Gynaecologists and Obstetricians of Burkina Faso in International Journal of Gynecology and Obstetrics, vol.127: S40-S42.

**31. Papp**, S.A., Gogoi, A., Campbell, C. (2013) Improving maternal health through social accountability: A case study from Orissa, India In Global Public Health, 8:4: 449-464.

**32. Pattinson**, R., Kerber, K., Waiswa, P., Day, L.T., Mussell, F., Asiruddin, S., Blencowe, H., Lawn, J.E. (2009) Perinatal mortality audit: Counting, accountability and overcoming challenges in scaling up in low-and middle-income countries in International Journal of Gynecology and Obstetrics, Vol.107: S113-S122.

**33. Penas De Fago** , M.A., Moran Faundes, J.M., (2014) Conservative litigation against sexual and reproductive health policies in Argentina in Reproductive Health Matters, Vol.22, no.44: 82-90.

**34. Rinker**, C.H. (2015) Creating Neoliberal Citizens in Morocco: Reproductive Health, Development Policy and Popular Islamic Beliefs in Medical Anthropology, 34: 226-242.

**35. Rosen**, H.E., Lynam, P.F., Carr, C., Reis, V., Ricca, J., Bazant, E.S., Bartlett, L.A. (2015) Direct observation of respectful maternity care in five countries: a cross-sectional study of health facilities in East and Southern Africa in BMC Pregnancy & Childbirth, Vol.15: 306.

**36. Scott**, H., Danel, I. (2016) Accountability for Improving Maternal and Newborn health in Best Practice & Research Clinical Obstetrics and Gynaecology, Vol.36: 45-56.

**37. Seelinger**, K.T. (2014) Domestic accountability for sexual violence: The potential of specialized units in Kenya, Liberia, Sierra Leone and Uganda. International Review of the Red Cross, Vol.96, no.804: 539-564.

**38. Shayo**, E.H., Mboera, L.E.G., Blystad, A. (2013) Stakeholders’ participation in planning and priority setting in the context of a decentralised health care system: the case of prevention of mother to child transmission of HIV programme in Tanzania in BMC Health Services Research, Vol.13: 273.

**39. Topp**, S.M., Black, J., Morrow, M., Chipukuma, J.M., Van Damme, W. (2015) The impact of human immunodeficiency virus (HIV) service scale-up on mechanisms of accountability in Zambian primary health centres: a case-based health systems analysis in BMC Health Services Research, Vol.15: 67.

**40. Tromp**, N., Prawiranegara, R., Riparev, H.S., Siregar, A., Sunjaya, D., Baltussen, R. (2015) Priority setting in HIV/AIDS control in West Java Indonesia: an evaluation based on the accountability for reasonableness framework in Health Policy and Planning, Vol.30: 345-355.
