# Supplementary material for: Broadening understanding of accountability ecosystems in sexual and reproductive health and rights: A systematic review
Source: PLoS One. 2018 May 31;13(5):e0196788. doi: 10.1371/journal.pone.0196788 (PMC5978882; doi:10.1371/journal.pone.0196788)
Supplement: S5 Table — (DOCX) [file pone.0196788.s005.docx]

**S5 - Summary of reported outcomes by type**

| **Type of outcome** | **Authors** |
| --- | --- |
| **Health outcomes** |  |
| Reduction peri-natal mortality after introduction of audit | Pattinson et al., 2014 [15] |
| **Contributing to health outcomes as part of a multi-pronged intervention** |  |
| Reduction in maternal mortality ratio following creation of pregnancy surveillance and registry system involving community-based health workers | Labrique et al., 2012 [33] |
| **Intermediary outcomes** |  |
| **Capabilities of rights-holders** |  |
| Women’s capacity to demand accountability improved | Papp et al., 2013 [18] |
| HIV patients empowered (but no improvement health worker attitudes) | Topp et al., 2015 [24] |
| **Provider practices** |  |
| Improvement in professional practice of providers | Papp et al., 2013 [18] |
| Improvement in the professional practice of health workers. | Hussein et al., 2012 [30] |
| **Health system level outcomes** |  |
| Increased uptake of MDSR committees, national confidential enquiry systems and the creation of national maternal death review committees in LMIC | Mathai et al., 2015 [29], Scott & Danel, 2016 [31] |
| Implementing audits in resource-constrained settings is achievable in terms of cost. The major cost is related to time to collect and analyze data. | Hussein et al., 2014 [30], Pattinson et al., 2014 [15] |
| **Changes in legislation, policies and guidelines including implementation of legislation, policies and guidelines** |  |
| Change in gender-related laws in Nepal and Sri Lanka following civil society action | Barrow, 2009 [36] |
| Court ordered the implementation of the National Rural Health Mission as a result of strategic litigation related to maternal death of poor woman | Kaur, 2012 [42] |
| **Unintended effects** |  |
| Religiously affiliated NGOs use strategic litigation to repeal implementation of progressive laws and policies | McCrudden, 2015 [41], Penas Defago et al., 2014 [39] |
| The state can use advances in SRHR policy to hide lack of progress in other ESR obligations. | Lind & Keating, 2013 [51] |
| Accountability strategies had a negative impact on quality of care for patients in need of other health services | Topp, et al., 2015 [57] |
